# Posttraumatic levels of liver enzymes can reduce the need for CT in children: a retrospective cohort study

**DOI:** 10.1186/s13049-016-0297-1

**Published:** 2016-08-25

**Authors:** Peter James Bruhn, Lene Østerballe, Jens Hillingsø, Lars Bo Svendsen, Frederik Helgstrand

**Affiliations:** 1 Department of Surgical Gastroenterology, Rigshospitalet, University of Copenhagen, Blegdamsvej 9, 2100 Copenhagen Ø, Denmark; 2 Department of Surgical Gastroenterology, Køge Hospital, University of Copenhagen, Lykkebækvej 1, 4600 Køge, Denmark

**Keywords:** Pediatric liver injury, Liver transaminases, Computed tomography, Blunt liver injury

## Abstract

**Background:**

Computed tomography (CT) is the gold standard in the initial evaluation of the hemodynamically stable patient with suspected liver trauma. However, the adverse effects of radiation exposure are of specific concern in the pediatric population. It is therefore desirable to explore alternative diagnostic modalities. Aspartate aminotransferase (AST) and alanine aminotransferase (ALT) are hepatic enzymes, which are elevated in peripheral blood in relation to liver injury. The aim of the present study was to investigate a potential role of normal liver transaminase levels in the decision algorithm in suspected pediatric blunt liver trauma.

**Methods:**

Retrospective analysis of consecutively collected data from children (0–17 years) with blunt liver trauma, admitted to a single trauma centre in Denmark, between 2000 and 2013. Patients underwent abdominal CT during initial evaluation, and initial AST and/or ALT was measured. Based on local guidelines, we set the threshold for blood AST and ALT level to 50 IU/L. Nonparametric statistical tests were used.

**Results:**

Sixty consecutive children with liver injury following blunt abdominal trauma were enrolled in the study. All patients with normal AST and/or ALT level were treated conservatively with success. Information on both AST and ALT was available in 47 children. Of these 47 children, three children had AST and ALT levels ≤50 IU/L. These children suffered from grade I liver injuries, and were treated conservatively with no complications.

**Discussion:**

All children who presented with blunt liver injury and AST and ALT levels ≤50 IU/L did not require treatment. These findings indicate that AST and ALT could be included in an updated management algorithm as a screening method to avoid abdominal CT. Notable limitations to the study was the retrospective method of data collection, without inclusion of a control group.

**Conclusions:**

CT seems superfluous in the initial evaluation of hemodynamically stable children with suspected blunt liver injury and blood AST and ALT levels ≤50 IU/L.

## Background

Blunt liver trauma is one of the most common and serious abdominal injuries [1]. Persisting hemodynamic instability in these patients, in spite of resuscitation attempts, should lead to surgical intervention [2]. However, the hemodynamically stable patient with no signs of other intraabdominal injury is usually treated conservatively, as is the case in 85 % of blunt liver injuries [2–6]. In some cases, in the hemodynamically stable patients with hepatic bleeding, surgery may yet be avoided, by arterial embolization [7].

Computed tomography (CT) of the abdomen is the gold standard in the initial evaluation of the hemodynamically stable blunt trauma patient, because it displays optimal sensitivity and specificity in diagnosing parenchymal injuries [8, 9]. Emergency CT is expensive and offers several limitations, including: risk of contrast agent allergy, contrast nephropathy, possible need for sedation, and is time consuming [9, 10]. Additionally, each abdominal CT increases the lifetime risk of cancer by 0.18 % in 1-year-old infants [9, 11]. Thus, it is desirable to find other diagnostic tools in the initial evaluation of the pediatric liver trauma patient [12, 13].

Transaminases are mitochondrial and cytoplasmic enzymes that are found in hepatocytes, neurons, pancreatic and muscle cells [14, 15]. The two most common transaminases are aspartate aminotransferase (AST) and alanine aminotransferase (ALT). Raised blood levels of AST and ALT are found when hepatocytes are damaged from inflammation, infection, trauma or surgical intervention [14–20]. AST and ALT elevation has been known to correlate to liver injury, and have been shown to occur immediately after the trauma [16–20]. Furthermore, it has been shown that high-grade (AAST grades III-VI) liver injury results in higher AST and ALT levels than low-grade liver injury (AAST grades I-II) [5, 11]. One study has indicated that ALT is most ideal to detect liver injury, compared to AST and other haematological markers [11].

Several studies have investigated the role of liver transaminase levels and FAST (focused assessment sonography in trauma) in initial diagnostic management of pediatric blunt liver traumas [1, 21–27]. The studies have found that liver transaminase levels as well as FAST are valuable screening tools in the decision algorithm in blunt liver injury. The studies, however, vary in focus and inclusion criteria. Thus, no consensus has been reached with regard to inclusion of liver enzymes in a specific decision algorithm.

The present study investigates whether liver transaminase levels are of value in the decision algorithm in pediatric blunt liver trauma, as a screening method to avoid emergency CT.

## Methods

We retrospectively analyzed consecutively registered data in a local database from all children (0–17 years) with blunt liver injury, who were initially admitted to the same level 1 trauma centre in Denmark, between 2000 and 2013. Data from the database were supported by data from patient files.

We included patients, who underwent abdominal CT during initial evaluation, and in whom initial AST and/or ALT were measured.

Outcomes were: age, gender, blood AST level, blood ALT level, FAST result, CT-diagnosed liver injury grade, management approach and presence of CT-diagnosed free fluid. Liver injury grade was classified by CT according to the AAST scale [5].

Before study analyses, based on local guidelines, we set the threshold for blood AST and ALT upper margin normal reference range level to 50 IU/L.

This study was exempt from approval by the Danish Capital Region Research Ethics Committee.

### Statistical analysis

Continuous variables were presented with median and interquartile range (IQR) and compared by the Mann-Whitney *U* test. Continuous variables in three or more groups were compared by the Kruskal-Wallis H test. Nominal variables were presented as observed frequencies and percentages and compared by Fisher’s exact test. The level of significance was defined as *p* < 0.05. SPSS v19.0 for Mac was used for data analysis.

## Results

From 2000 to 2013, 117 children were admitted to our level 1 trauma centre with blunt liver injury. In total, 57 patients were excluded due to missing CT (*n* = 26) and AST or ALT (*n* = 31). Thus, in total, 60 children with liver injury following blunt trauma were enrolled in the study. Patient characteristics are presented in Table 1.

**Table 1:** General characteristics of the study population

Age, median (IQR^a^), years	11 (7–15)
Gender, *n* (%)	
Female	21 (35)
Male	39 (65)
FAST^b^, *n* (%)	
Positive	32 (65.3)
Negative	17 (34.7)
AAST liver injury grade, *n* (%)	
Grade 1	5 (8.3)
Grade 2	18 (30)
Grade 3	22 (36.7)
Grade 4	13 (21.7)
Grade 5	2 (3.3)
Grade 6	0 (0.0)
Free fluid in CT^c^, *n* (%)	
Present	44 (73.3)
Absent	16 (26.7)
AST^d^ and/or ALT^e^ level > 50 IU/L, *n* (%)	
Present	44 (93.6)
Absent	3 (6.4)

^a^Interquartile range, ^b^focused assessment sonography in trauma, ^c^computed tomography, ^d^aspartate aminotransferase, ^e^alanine aminotransferase

Blood AST and ALT were measured in 48 and 59 patients, respectively, and showed significant positive correlation to liver trauma grade (*p* = 0.002 for AST, and *p* = 0.022 for ALT), Table 2. AST as well as ALT levels were significantly higher in patients with high-grade (AAST grades III-VI) liver injury (*p* < 0.001 for AST, and *p* = 0.003 for ALT), compared to patients with low-grade liver injury (AAST grades I-II).

**Table 2:** Liver enzyme levels in relation to liver injury grade

Blood AST^a^ level (*n* = 38), median (IQR^b^), IU/L	379 (140–570.25)
Blood ALT^c^ level (*n* = 48), median (IQR), IU/L	303 (139–476)
AST level, median (IQR)	
Grade 1	36 (29.5–225.5)
Grade 2	136 (95–375.5)
Grade 3	515 (405.75–688.5)
Grade 4	451 (283.75–642)
Grade 5	462 (270–462)
Grade 6	-
ALT level, median (IQR)	
Grade 1	21 (13–191)
Grade 2	239 (124–432)
Grade 3	438 (230.5–501.75)
Grade 4	351 (255–492)
Grade 5	307.5 (285–307.5)
Grade 6	-

^a^aspartate aminotransferase, ^b^interquartile range, ^c^alanine aminotransferase

Both enzymes were measured in 47 cases. No cases were observed where an elevated AST was not accompanied by elevated ALT, or vice versa.

Descriptions of FAST were available in 49 cases, 32 of which were positive. Two patients presented with positive FAST and AST and/or ALT ≤ 50 IU/L. Positive FAST results did not show significant correlation to liver injury grade (*p* = 0.716). Furthermore, positive FAST results did not show significant correlation to whether patients were managed conservatively, by arterial embolization or by surgery (*p* = 0.997).

Liver enzyme levels > 50 IU/L did not show correlation to management approach (*p* = 0.755), as presented in Table 3.

**Table 3:** Liver enzyme levels in relation to management approach

	Conservative treatment	Surgery	Arterial embolization	Total
AST^a^ and ALT^b^ ≤ 50 IU/L	3	0	0	3
AST and/or ALT > 50 IU/L	37	3	4	44
Total	40	3	4	47

^a^aspartate aminotransferase, ^b^alanine aminotransferase

Of the 47 patients, where both liver enzyme levels were measured and CT undertaken, three (6.4 %) children with grade I liver injuries presented with AST and ALT equal to or below 50 IU/L. These children were treated conservatively and were discharged after 0 (<24 h), 1 and 3 days, respectively. There were no following complications in either case. Two of these patients initially presented with positive FAST.

All patients with AST and/or ALT ≤ 50 IU/L (*n* = 3) were treated conservatively with success. In the 47 patients, where both AST and ALT was available, invasive treatment was performed in seven cases, either in the form of arterial embolization (*n* = 4) or surgery (*n* = 3). In all of these cases, the child had presented with AST as well as ALT levels above 250 IU/L. Of the conservatively treated patients, 60.4 % (*n* = 32/53) presented with AST and/or ALT levels above 250 IU/L.

## Discussion

In this cohort of retrospectively analysed pediatric CT verified blunt liver injuries, all but three children initially presented with raised AST and/or ALT levels (>50 IU/L). These children’s liver injuries did not require treatment. All patients with normal levels of AST and/or ALT underwent conservative treatment. In addition, we found a significant correlation between AST and/or ALT levels and grade of liver injury determined by AAST levels on CT. AST and ALT were equally reliable in detecting liver injury.

**Fig. 1:**
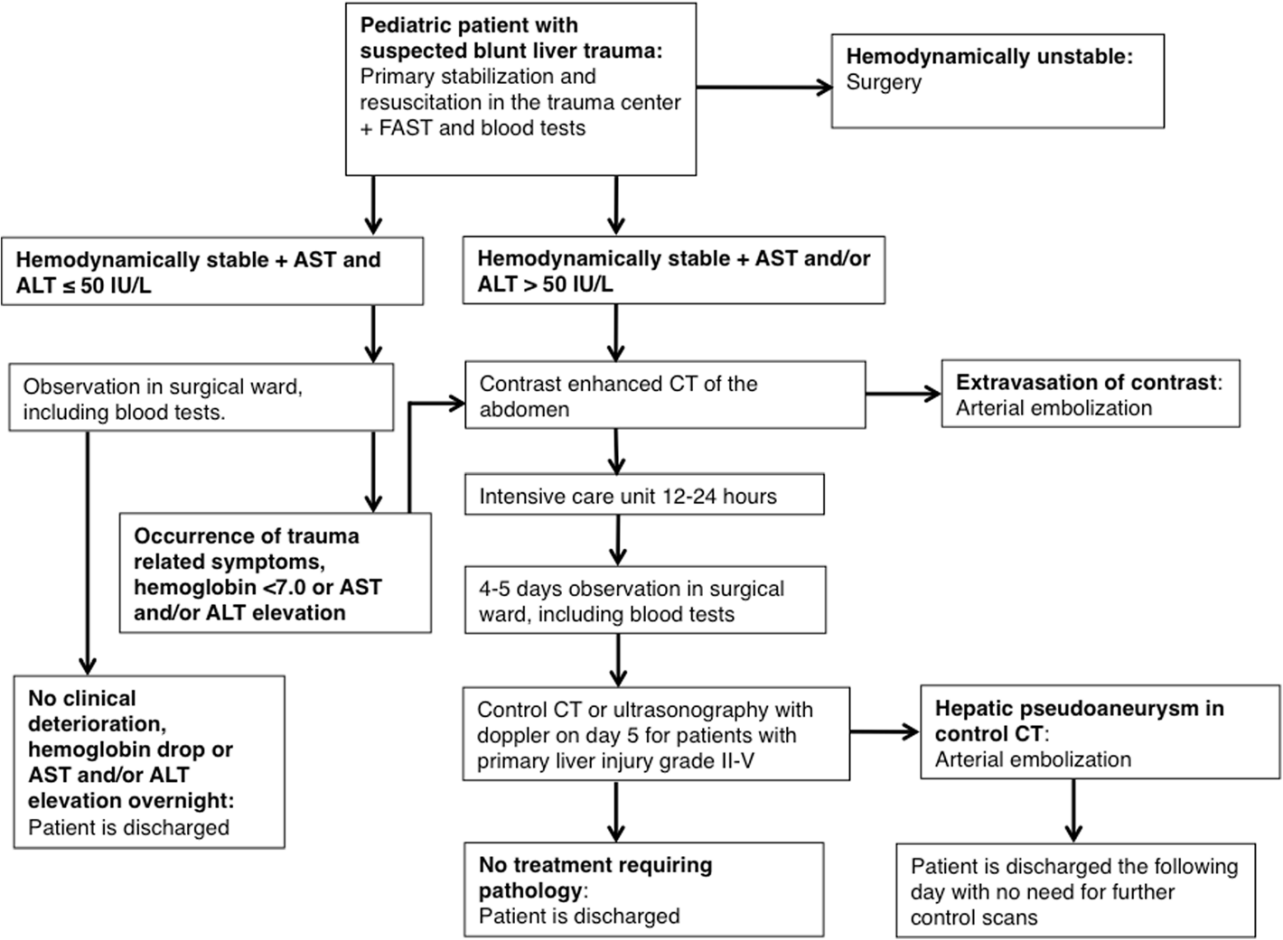
Revised algorithm for evaluation and treatment of pediatric blunt liver injury

In many centres, contrast enhanced CT of the abdomen is presently mandatory in the diagnosis and treatment algorithm of a hemodynamically stable pediatric patient with suspected liver injury following relevant blunt abdominal trauma [28–30]. In agreement with others, we found that initial evaluation of AST and ALT might be a useful diagnostic tool to predict the need for CT. This could result in time, cost and safety benefits in relation to the initial evaluation of hemodynamically stable patients with potential blunt liver injury [31]. On the basis of our results, we propose a revised algorithm for management of pediatric blunt liver injury, which includes evaluation of AST and ALT as a screening method to avoid abdominal CT (Fig. 1). The revised algorithm introduces a possibility to omit immediate abdominal CT scan in the case of a hemodynamically stable child without abdominal pain, along with normal liver enzyme levels, leading to immediate transfer to the surgical ward. We propose an overnight observation period for these children based on the results of St Peter et al. in a prospective study [6]. It should be emphasized that any clinical suspicion of treatment requiring trauma, should lead to immediate radiological examination.

We applied an upper reference value of AST and ALT of 50 IU/L based on local guidelines. Clinically applied reference ranges for AST and ALT varies with sex and age [32], and therefore we set the upper reference value for both enzymes to a common value well above the locally applied values for children of both sexes. Future studies may find that our threshold is too conservative, and if so, the use of emergency CT could be reduced even more.

Several studies have been performed on the diagnostic capability of evaluation of various threshold levels for AST and ALT [1, 21–27]. The previous studies analysed pediatric patient data, but had varying focus concerning primary condition leading to CT, whether or not FAST was performed, and which upper liver transaminase reference value was applied. Overall, our results are supported by these previous studies, which indicated that evaluation of liver enzyme levels could indeed be applied as an argument to abstain from emergency abdominal CT [1, 21–27]. Some of these studies showed remarkably high sensitivity and specificity in diagnosing blunt liver injury with application of varying liver enzyme reference ranges [21–23].

Our study is consistent with the existing literature in the context that the presence of significant liver injury can indeed be expected to result in raised liver enzyme levels.

We did not find significant correlation between FAST result and liver injury grade or management approach. However, FAST is easy to repeat and does play an integral role in the evaluation of blunt traumas suspected for bleeding [33].

In our cohort, 6.4 % of the emergency CT scans could have been omitted by application of AST and ALT levels >50 IU/L as a screening method. However, our cohort consisted exclusively of patients with liver trauma admitted to a single level 1 trauma center, where pre-hospital selection was applied. It can be assumed that the proportion of patients with non-treatment requiring liver injuries and low liver enzyme levels, will be higher in primary centers, where wider pre-hospital selection criteria are applied. Therefore, it is our assumption that in the situation where the only indication for emergency CT scan is the suspicion of liver injury, considerably more than 6.4 % of these scans can be avoided by application of the mentioned screening method. Naturally, there is a risk of missing other organ injury; the extent and consequences of these missed injuries can only be speculated, and will probably be negligible after repeated clinical examinations. Specifically, St Peter et al. showed that an abbreviated bedrest protocol is safe in the case of low-grade blunt liver and spleen injury, without risk of complications after discharge [6].

A notable limitation of our study was in the method of data collection. Data was collected retrospectively through a local database and supported with data from patient files. Furthermore, it was not possible to obtain data on the registration rate in the database. This led to exclusion of 57 out of 117 patients in our study. It can only be speculated if the possible missed inclusions have led to selection bias. The study was conducted without inclusion of a control group. Finally, in 13 patients we only had data on either AST or ALT levels. Due to the mentioned limitations present results need to be confirmed in a prospective study setting.

## Conclusions

In conclusion, the present study supports that emergency abdominal CT for suspected blunt liver injuries in hemodynamically stable and unaffected children can be omitted in the presence of normal liver enzyme levels, provided that no other indication for emergency CT is present. The children should be admitted for observation in the ward, and undergo CT if trauma related symptoms occur, intensify, or a later increase in AST and/or ALT is found.
